# Statin-activated nuclear receptor PXR promotes SGK2 dephosphorylation by scaffolding PP2C to induce hepatic gluconeogenesis

**DOI:** 10.1038/srep14076

**Published:** 2015-09-22

**Authors:** Saki Gotoh, Masahiko Negishi

**Affiliations:** 1 Pharmacogenetics Section, Reproductive and Developmental Biology Laboratory, National Institute of Environmental Health Sciences, National Institutes of Health, Research Triangle Park, North Carolina 27709, USA

## Abstract

Statin therapy is known to increase blood glucose levels in humans. Statins utilize pregnane X receptor (PXR) and serum/glucocorticoid regulated kinase 2 (SGK2) to activate phosphoenolpyruvate carboxykinase 1 (*PEPCK1*) and glucose-6-phosphatase (*G6Pase*) genes, thereby increasing glucose production in human liver cells. Here, the novel statin/PXR/SGK2-mediated signaling pathway has now been characterized for hepatic gluconeogenesis. Statin-activated PXR scaffolds the protein phosphatase 2C (PP2C) and SGK2 to stimulate PP2C to dephosphorylate SGK2 at threonine 193. Non-phosphorylated SGK2 co-activates PXR-mediated trans-activation of promoters of gluconeogenic genes in human liver cells, thereby enhancing gluconeogenesis. This gluconeogenic statin-PXR-SGK2 signal is not present in mice, in which statin treatment suppresses hepatic gluconeogenesis. These findings provide the basis for statin-associated side effects such as an increased risk for Type 2 diabetes.

The nuclear receptor PXR was originally characterized as a drug-sensing transcription factor that activates genes involved in drug metabolism such as cytochrome P450 (*CYP*) genes^1^. Subsequent investigations have greatly widened the biological roles of PXR from drug metabolism to various other types of liver functions, one of which is regulation of energy metabolism including hepatic gluconeogenesis^2^,^3^,^4^. Activation of PXR by therapeutics resulted in the suppression of hepatic gluconeogenesis and decreased blood glucose levels in mice^3^,^4^. Activated PXR suppressed transcription of *PEPCK1* and *G6Pase* genes by binding transcription factor Forkhead box protein O1 (FOXO1)^4^ or by binding CRE (cAMP-response element)-binding protein (CREB)^5^ in mouse livers. Peroxisome proliferator-activated receptor γ coactivator 1 α was also suggested to repress PEPCK1 through inteaction with HNF4α in HepG2 cells^6^. However, the suggested mechanism was primarily extablished by tageting two *CYP* genes *CYP7A1* and *CYP8B1*. However, contrary to what was observed in mice, our recent study demonstrated that treatment with statins or rifampicin activated these gluconeogenic genes and increased glucose production in human liver cells^7^. FOXO1 was not involved in this process, but PXR required SGK2 (the predominant member of the SGK kinase family found in the liver^8^) for activation of gluconeogenic genes. siRNA knockdown of SGK2, in fact, severely attenuated the rifampicin-induced transcription in human liver cells^7^. This finding of drug-induced gluconeogenesis is consistent with clinical observations that treatment with statins or rifampicin can increase blood glucose levels in humans^9^,^10^,^11^,^12^. With regard to statin therapy, new-onset diabetes has been observed in clinical trials and meta-analysis^13^, forcing the Food and Drug Administration to issue a new safety warning noting that statins can increase the risk of developing Type 2 diabetes. However, the molecular mechanism of this side effect still remains unresolved, although several possibilities have been proposed^14^. In attempt to provide the basis of drug-induced gluconeogenesis, here we have determined the molecular mechanism by which PXR utilizes SGK2 to activate gluconeogenic genes following simvastatin treatment.

## Results

### PXR and SGK2 in statin induction

**Figure 1:**
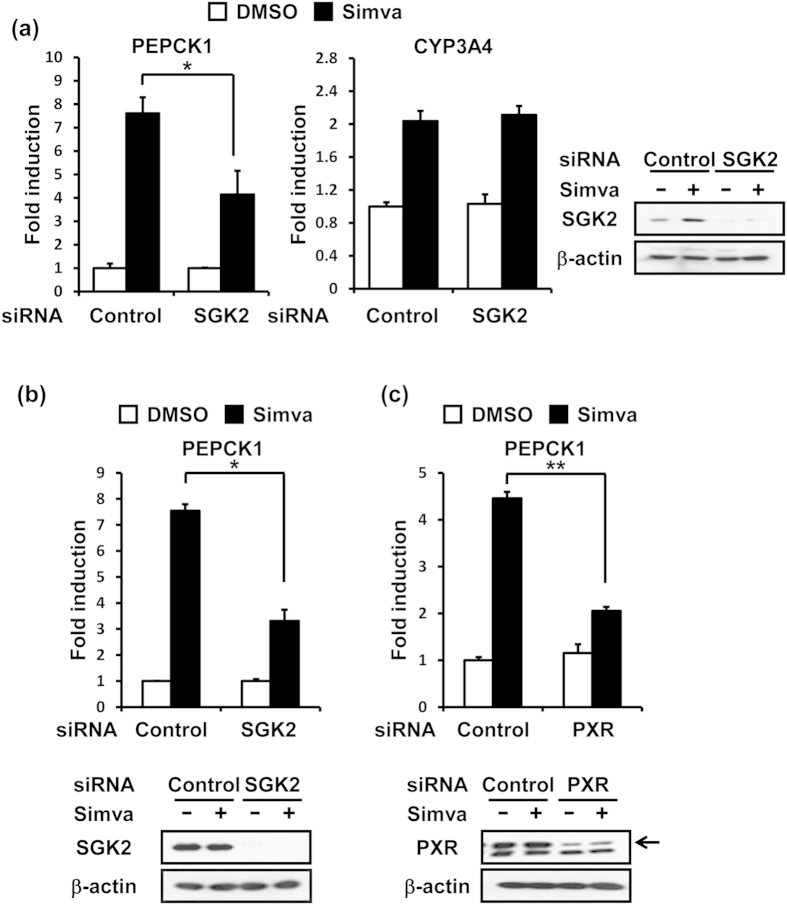
Statin induction of the *PEPCK1* gene mediated by SGK2 and PXR. (**a**) Relative expression of PEPCK1 (left) and CYP3A4 (middle) mRNA levels measured by qRT-PCR in SGK2 siRNA-transfected human primary hepatocytes treated with simvastatin (Simva, 10 μM) for 24 h. Results are shown as fold change relative to DMSO treated cells. (n = 3, mean ± s.d) *P < 0.05, determined by, Student’s t test. Right, Western blot analysis of SGK2 and β-actin from whole cell lysates of each of these human primary hepatocytes. (**b**,**c**) Top, relative expression of PEPCK1 mRNA levels measured by qRT-PCR in SGK2 siRNA or PXR siRNA-transfected ShP51 cells treated with simvastatin (Simva, 10 μM) for 2.5 and 5 h. Results are shown as fold change relative to DMSO treated cells (n = 3, mean ± s.d) *P < 0.05, **P < 0.01, determined by Student’s t test. Bottom, Western blot analysis of SGK2, PXR and β-actin from whole cell lysates of each of these ShP51cells.

SGK2 siRNA attenuated the simvastatin-induced increase of PEPCK1 mRNA from 8- to 4-fold in human primary hepatocytes, while simvastatin induction of CYP3A4 mRNA, a classic target of PXR, remained unchanged (Fig. 1a). Thus, SGK2 was involved in simvastatin-induced activation of the *PEPCK1* gene but not *CYP3A4* gene. Similar regulation by SGK2 was also observed in human hepatoma HepG2 cell-derived ShP51 cells that stably express human PXR^15^ (Fig. 1b). In addition, treatment with PXR siRNA confirmed that the simvastatin-induced activation of the *PEPCK1* gene required both PXR and SGK2 (Fig. 1c). In addition to PEPCK1, simvastatin induction of G6Pase mRNA was similarly regulated by both PXR and SGK2 in human primary hepatocytes as well as ShP51 cells (Supplementary Fig. 1a,b). Therefore, ShP51 cells retain the ability of hepatocytes respond to simvastatin.

### SGK2 dephosphorylation at Thr193

**Figure 2:**
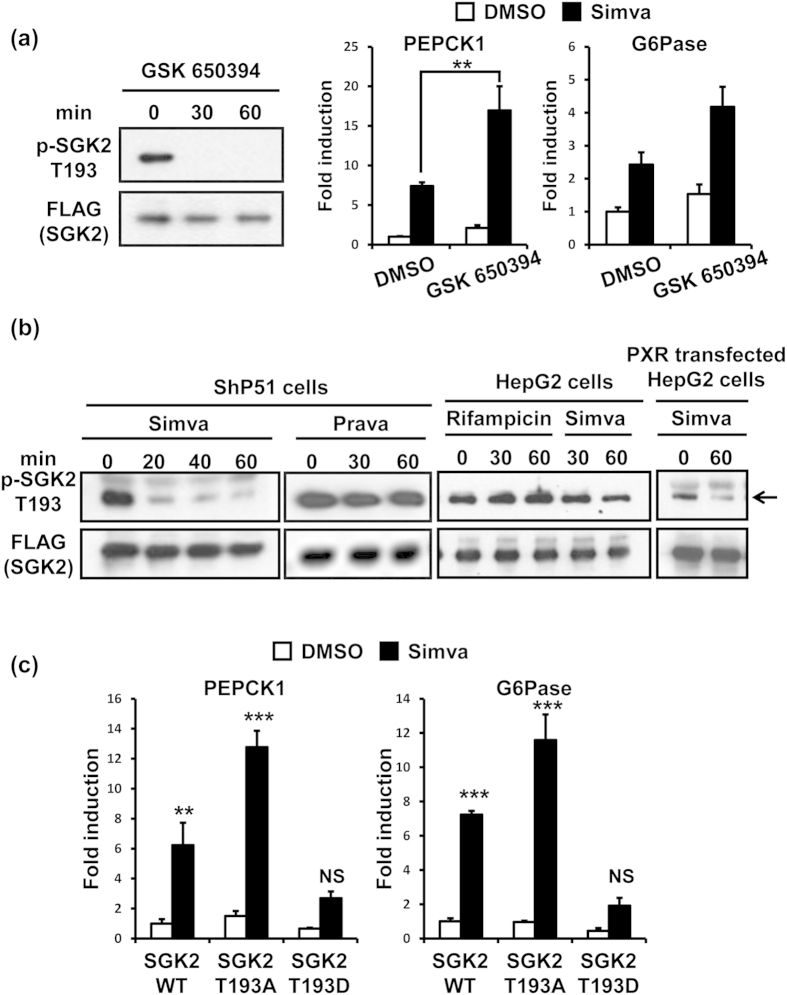
Statin activated PXR-dependent SGK2 dephosphorylation. (**a**) Left, Western blot analysis of immunoprecipitated p-SGK2 T193 and FLAG-SGK2 from whole cell lysates in pcDNA/FLAG/SGK2-transfected ShP51 cells treated with GSK650394 (10 μM) for 30 and 60 min. Right, relative expression of PEPCK1 and G6Pase mRNA levels measured by qRT-PCR in ShP51 cells pretreated with GSK650394 (10 μM) for 1 h, followed by co-treatment with simvastatin (Simva, 10 μM) for additional 3 h. Results are shown as fold change relative to DMSO treated cells (n = 3, mean ± s.d). (**b**) Western blot analysis of immunoprecipitated p-SGK2 T193 and FLAG-SGK2 from whole cell lysates in pcDNA/FLAG/SGK2-transfected ShP51 cells treated with simvastatin (Simva, 10 μM) or pravastatin (Prava, 10 μM) for 20, 30, 40 and 60 min (Left), in pcDNA/FLAG/SGK2-transfected HepG2 cells treated with rifampicin (100 μM) or simvastatin (Simva, 10 μM) for 30 and 60 min (middle), and in pcDNA/FLAG/SGK2 and pCR3/PXR-transfected HepG2 cells treated with simvastatin (Simva, 10 μM) for 60 min (right). Data shown were generated from , at least, three indsepedent experiments. (**c**) Relative expression of PEPCK1 and G6Pase mRNA levels measured by qRT-PCR in ShP51 cells which were transfected with pcDNA/SGK2 wild type (WT), pcDNA/SGK2 T193A or pcDNA/SGK2 T193D after endogenous SGK2 was knocked down by siRNA, followed by treatment with simvastatin (Simva, 10 μM) for 3 h. Results are shown as fold change relative to DMSO treated SGK2 WT-transfected cells (n = 3, mean ± s.d) *P < 0.05, **P < 0.01, ***P < 0.001. NS, not significant by One-way ANOVA analysis.

SGK2 phosphorylation at threonine 193 (Thr193)^8^ was examined in order to understand how SGK2 regulated simvastatin-induced transcription of gluconeogenic genes. Ectopically expressed SGK2 was phosphorylated at Thr193 and was dephosphorylated following treatment with a SGK2 inhibitor GSK650394^16^ in ShP51 cells. This dephosphorylation correlated with the increases of PEPCK1 and G6Pase mRNAs at both basal and simvastatin-induced levels (Fig. 2a). Thus, dephosphorylation at Thr193 apparently enabled SGK2 to activate gluconeogenic genes. Since simvastatin activated PXR to engage SGK2-mediated induction of glucogenogenic genes, whether or not SGK2 dephosphorylation depended on simvastatin-activated PXR was examined. It was, in fact, simvastatin and not pravastatin (non-PXR activator^17^,^18^) that stimulated the dephosphorylation of Thr193 in ShP51 cells (Fig. 2b). HepG2 cells with very low levels of PXR^5^ could not dephosphorylate Thr193 even after treatment with rifampicin or simvastatin, whereas overexpression of human PXR enabled this response (Fig. 2b). These results indicate that SGK2 underwent an activated PXR-dependent dephosphorylation at Thr193. Next, the effects of this dephosphorylation on the ability of SGK2 to regulate simvastatin-induced increase of PEPCK1 and G6Pase mRNAs were examined. Wild type SGK2, phosphorylation-mimicking SGK2 T193D or non-phosphorylation-mimicking SGK2 T193A was overexpressed in ShP51 cells after endogenous SGK2 was knocked down by SGK2 siRNA. SGK2 T193A-expressing cells most effectively responded to simvastatin and increased both PEPCK1 and G6Pase mRNAs (Fig. 2c). On the other hand, overexpression of SGK2 T193D severely attenuated simvastatin-induced as well as decreased basal mRNA levels. Thus, PXR-mediated dephosphorylation of Thr193 was required for SGK2 to increase these mRNAs in response to simvastatin.

### PP2Cα-mediated dephosphorylation of SGK2

**Figure 3:**
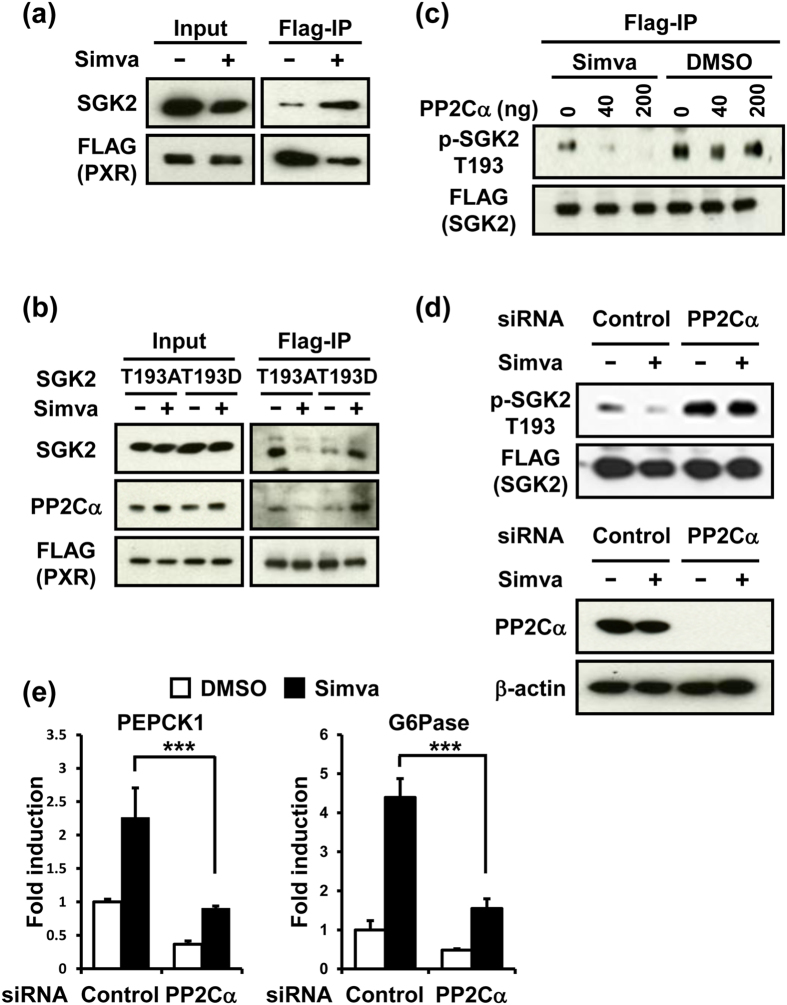
PP2Cα-mediated dephosphorylation of SGK2. (**a**) Immunoprecipitation of FLAG-PXR and Western blot analysis of SGK2 and FLAG-PXR in pCR3/FLAG/PXR-transfected ShP51 cells treated with simvastatin (Simva, 10 μM) for 60 min. (**b**) Immunoprecipitation of FLAG-PXR and Western blot analysis of SGK2, PP2Cα and FLAG-PXR in pCR3/FLAG/PXR and pcDNA/SGK2 T193A or pcDNA/SGK2 T193D-transfected ShP51 cells treated with simvastatin (Simva, 10 μM) for 60 min. (**c**) The dephosphorylation of SGK2 at Thr193 assessed by *in vitro* dephosphorylation assay using pure PP2Cα as an enzyme and FLAG-immunoprecipitated SGK2 in pcDNA/FLAG/SGK2-transfected ShP51 cells treated with simvastatin (Simva, 10 μM) for 60 min as a substrate. (**d**) Top, Western blot analysis of immunoprecipitated p-SGK2 T193 and FLAG-SGK2 from whole cell lysates in PP2Cα siRNA-transfected ShP51 cells treated with simvastatin (Simva, 10 μM) for 45 min. Bottom, Western blot analysis of PP2Cα and β-actin from whole cell lysates of each of these ShP51cells. Data shown were generated from three independent experiments. (**e**) Relative expression of PEPCK1 and G6Pase mRNA levels measured by qRT-PCR in PP2Cα siRNA-transfected ShP51 cells treated with simvastatin (Simva, 10 μM) for 3 h. Results are shown as fold change relative to DMSO treated control siRNA transfected cells. (n = 3, mean ± s.d) ***P < 0.001. NS, not significant, determined by One-way ANOVA.

Various protein phosphatase inhibitors were examined for their effects on simvastatin-induction of PEPCK1 and G6Pase mRNAs levels in ShP51 cells. Only sanguinarine chloride, but not okadaic acid, tautomycin or fostriecin, inhibited these increases (Supplementary Fig. 2a–c). Sanguinarine chloride preferentially inhibits protein phosphatase 2C (PP2C)^19^, while the others are either PP1 and/or PP2A inhibitors. Co-immuoprecipitation assays indicated that SGK2 interacted with PP2Cα but not either PP1α or PP2A (Supplementary Fig. 2d). While the interaction between SGK2 and PP2Cα appeared to be constitutive, the interaction of SGK2 with PXR was enhanced in response to simvastatin treatment (Fig. 3a). Consistent with these two observations, simvastatin treatment elicited an interaction between PXR and the SGK2 T193D/PP2Cα complex (Fig. 3b). SGK2 T193A interacted with PXR and this interaction reduced after simvastatin treatment, the reason for which remains to be explored in a future study. *In vitro* dephosphorylation assays were reconstituted utilizing phosphorylated FLAG-tagged SGK2 immunoprecipitated from whole extracts of simvastatin- or DMSO-treated ShP51 cells as substrates and pure PP2Cα as the enzyme. PP2Cα dephosphorylated SGK2 at Thr193 from simvastatin-treated cells, but not from DMSO-treated cells (Fig. 3c). Thus, PP2Cα appears to be the phosphatase that dephosphorylates SGK2 and this dephosphorylation requires statin-activated PXR, likely as a scaffolding protein. PXR seems to require, at least, two separate regions (residues from 107/141 and from 334/348 of SGK2) for scaffolding SGK2 and PP2Cα (Supplementary Fig. 3). Moreover, siRNA knockdown of PP2Cα abolished simvastatin-induced SGK2 phosphorylation as well as severely attenuated the increase of PEPCK1 and G6Pase mRNAs in ShP51 cells (Fig. 3d,e).

### PXR and SGK2 interacting with promoters

**Figure 4:**
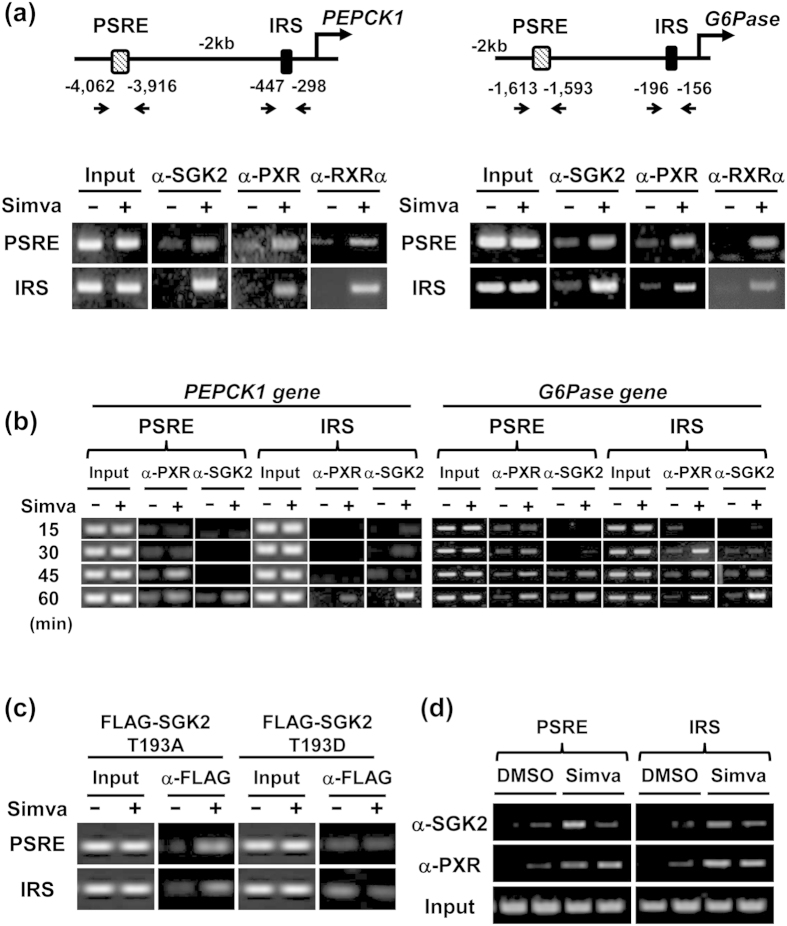
PXR-SGK2 responsiveness of the *PEPCK1* and *G6Pase* promoters. (**a**) Top, schematic representation of upstream region of the *PEPCK1* (left) and *G6Pase* (right) genes. Bottom, ChIP assays of SGK2, PXR and RXRα levels at PSRE (PEPCK1: −4,062/−3,916, G6Pase: −1,613/−1,593) and IRS (PEPCK1: −447/−298, G6Pase: −196/−156) regions in ShP51 cells treated with simvastatin (Simva, 10 μM) for 60 min (**b**) Time course ChIP assays of SGK2 and PXR levels at PSRE and IRS regions in ShP51 cells treated with simvastatin (Simva, 10 μM) for 15, 30, 45 and 60 min. (**c**) ChIP assays of SGK2 T193A and SGK2 T193Dlevels at PSRE and IRS regions in pcDNA/FLAG-SGK2 T193A or pcDNA/FLAG-SGK2T193D-transfected ShP51 cells treated with simvastatin (Simva, 10 μM) for 1 h. (**d**) ChIP assays of SGK2 and PXR levels at PSRE and IRS regions in human primary hepatocytes treated with simvastatin (Simva, 10 μM) for 3 h. Data shown were generated from three independent experiments.

A GCG SeqLab sequence homology search predicted five common PXR binding sequences conserved in both *PEPCK1* and *G6Pase* promoters up to −10 Kbp, and chromatin immunoprecipitation (ChIP) assays delineated a PXR binding site to D region (−1,613/−1,593) of the *G6Pase* promoter in ShP51 cells (Supplementary Fig. 4). This D region corresponds to the −4,062/−3,916 region within the *PEPCK1* promoter. Further ChIP analysis revealed both D regions interacting with SGK2, PXR and RXRα in response to simvastatin treatment; these regions are now referred to as the PXR-SGK2 response elements (PSRE). In addition to PSRE, a proximal region that includes an insulin response sequence (IRS) was also found to be essential for simvastatin-induced promoter activation in luciferase reporter assays (Supplementary Fig. 5). Simvastatin treatment also increased the interactions between IRS and factors SGK2, PXR and RXRα (Fig. 4a). These interactions indicated that both PXR and SGK2 function as transcription factors. PXR and SGK2 interacted with PSRE and IRS in a time-dependent manner in response to simvastatin (Fig. 4b). Within the *PEPCK1* promoter, the PXR was first recruited to the PSRE, followed by SGK2. Binding to the IRS lagged behind that of the PSRE; however in this instance PXR and SGK2 simultaneously interacted with the IRS. The *G6Pase* promoter slightly differed in its pattern of interactions: PXR interacted with the PSRE and IRS at the same time, followed by SGK2 interacting with both PSRE and IRS. Therefore, transcription appears to be initiated by interacting PSRE with a simvastatin-activated PXR-RXRα heterodimer and subsequently be co-activated by SGK2 interacting with both PSRE and IRS.

### Co-activation by non-phosphorylated SGK2 of PXR-mediated transcription

Simvastatin induced dephosphorylation of SGK2 at Thr193 and a subsequent increase of PEPCK1 and G6Pase mRNA levels (Fig. 2b,c). To examine whether or not dephosphorylation was essential for SGK2 to co-activate promoter, ChIP assays with *PEPCK1* promoter were performed in either SGK2 T193A- or SGK2 T193D- expressing ShP51 cells. Only SGK2 T193A, but not SGK2 T193D, interacted with PSRE and IRS when PXR was activated by simvastatin (Fig. 4c). Thus, dephosphorylation might enable SGK2 to co-activate the promoter. SGK2 is a member of the SGK family and shares structural features as protein kinase with AKT^8^. With this similarity, SGK2 may be activated by phosphorylation. However, these enzymatic properties of SGK2 have not been characterized at the moment. If, in fact, non-phosphorylated SGK2 is enzymatically inactive, SGK2 does not require its kinase activity to co-activate the promoter. On the other hand, SGK2 T193D mutant was found to interact with IRS before simvastatin treatment but it was dissociated after treatment. Therefore, phosphorylated form of SGK2 may also have its own function to regulate the promoter, the biology of which remains to be explored in the future investigations. We also confirmed that PXR and SGK2 interacted with both PSRE and IRS within the *PEPCK1* promoter in response to simvastatin treatment in human primary hepatocytes (Fig. 4d). Simvastatin treatment decreased hepatic PEPCK1 mRNA levels in mice and this decrease was associated with an apparent increase of SGK2 phosphorylation at Thr193 (Supplementary Fig. 6). Thus, this increased SGK2 phosphorylation might be a basis for the attenuated hepatic gluconeogenesis in mice after simvastatin treatment although the mechanism remains unknown at the moment. However, these distinct responses in phosphorylation of SGK2 may be the reason for species differences; humans increase gluconeogenesis while mice decrease it after statin treatment. SGK2 knockout mice will help us to better understand the SGK2-mediated regulation of hepatic gluconeogenesis.

**Figure 5:**
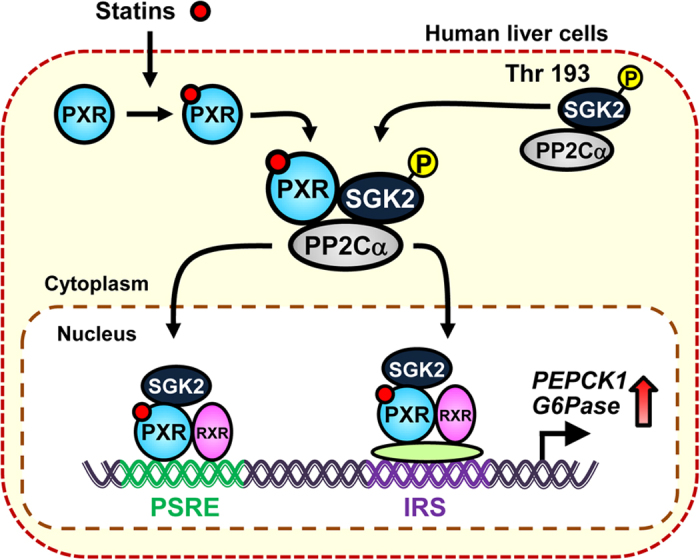
Schematic representation of the statin-PXR-SGK2 pathway. Statin-activated PXR binds to SGK2 phosphorylated at threonine 193 and facilitates dephosphorylation by PP2Cα. Subsequently, these PXR and dephosphorylated SGK2 bind PSRE and IRS within the promoter to activate it.

In conclusion, we have defined the molecular mechanism of statin-induced hepatic gluconeogenesis in human liver cells (Fig. 5). This induction involves three factors: PXR, SGK2 and PP2Cα. PXR has a dual function as signal scaffold stimulating dephosphorylation of SGK2 by PP2Cα as well as a transcription factor to trans-activate gluconeogenic gene promoters. The non-phosphorylated SGK2 co-activates PXR-mediated transcription. This drug-PXR-SGK2 pathway appears to be unique in human liver cells is not present in mouse livers. Although whether or not human liver cells utilize the previous described PXR-FOXO1 or CREB-mediated suppression found in mouse livers remains to be confirmed in future investigations, even if they did, the PXR-SGK2 pathway overwhelmed them to induce gluconeogenic genes. In addition, statins and insulin competed with each other to regulate gluconeogenic genes in ShP51 cells (unpublished information). Currently whether or not the PXR-SGK2-mediated guconeogenic signal can be regulated by physiological stimuli such as fasting and feeding is under investigation. There are numerous known single nucleotide polymorphisms in each of these genes in humans; 989, 766 and 1017 in the *PXR, SGK2* and *PP2C*α genes, respectively. Mutations in one of these genes or in various combinations with the other genes may increase a risk for side effects. This molecular mechanism of PXR-SGK2 signaling may help us to understand statin-caused side effects such as Type 2 diabetes and develop ways to prevent such adversities.

## Methods

All experimental protocols were approved by the National Institute of Environmental Health Sciences (NIEHS).

### Chemicals and reagents

Simvastatin, rifampicin, pravastatin sodium salt hydrate, phosphatase inhibitor cocktail 2, phosphatase inhibitor cocktail 3, ANTI-FLAG M2 Affinity Gel, ANTI-FLAG M2-Peroxidase (HRP) and monoclonal ANTI-FLAG M1 antibodies were purchased from Sigma-Aldrich (St. Louis, MO); restriction endonucleases and DNA-modifying enzymes from New England Biolabs (Beverly, MA); cOmplete Protease Inhibitor Cocktail Tablets (Roche, Basel, Switzerland); okadaic acid from EMD Millipore (Billerica, MA); fostriecin, sanguinarine chloride and GSK 650394 from Tocris Bioscience (Bristol, UK); anti-PXR antibody from Perseus Proteomics Inc. (Tokyo, Japan); anti-SGK2 antibody and anti-PP2Cα (D18C10) XP antibody from Cell Signaling (Danvers, MA); anti-PP2Ac antibody from BD Biosciences (Franklin Lakes, NJ); anti-p-SGK antibody (Thr256), anti-β-actin antibody (C4), PP1α (C-19) antibody, anti-RXR (ΔN 197) antibody. goat anti-rabbit IgG-HRP, goat anti-mouse IgG-HRP, donkey anti-goat IgG-HRP, normal mouse IgG and tautomycin from Santa Cruz Biotechnology (Santa Cruz, CA).

### Cells and culture

HepG2 cells and the HepG2-derived ShP51 cells stably expressed human PXR^15^ were cultured in minimum essential medium supplemented with 10% fetal bovine serum,100 units/ml penicillin, 100 μg/ml streptomycin and 2 mM L-glutamine. Human primary hepatocytes were obtained from Invitrogen (Carlsbad, CA) and cultured in Williams’ Medium E supplemented with Primary Hepatocyte Maintenance Supplements (Life Technology, Grand Island, NY).

### Animal experiments

C3H/HeNCrlBR male mice (Charles River), 9 weeks of age were maintained with 12 h light/12 h dark cycle (7:00 am to 7:00 pm). Mice were randomly housed into two groups (3 mice per group).100 μL of aqueous simvastatin (10% DMSO solution in PBS, 50 mg/kg b.wt.) was administrated to each of mice by oral gavage once daily for 2 days. After treatment, mice were sacrificed, and mice liver cDNAs and protein were prepared for qRT-PCR and Western blot respectively. Mouse livers were homogenized in 50 mM Tris-HCl buffered saline (pH 7.6) containing 8 M urea and 1% SDS and centrifuged. Proteins were analyzed by Western blot using anti-p-SGK2 antibody. Animals were handled according to NIEHS Animal Care and Use Committee guidelines and in compliance with NIEHS approved animal protocol.

### Plasmids

pCR3/PXR and pCR3/FLAG/PXR were described previously^5^. To construct pcDNA/SGK2, cDNA encoding the full-length human SGK2 isoform α was amplified with proper PCR primers (Supplementary Table 1) and was cloned into pCR2.1-TOPO (Invitrogen), from which the DNA insert was cut out by digestion with *Eco*RI and was inserted at the *Eco*RI site of pcDNA3.1 (Invitrogen). To construct pcDNA/hSGK2 T193A and pcDNA/SGK2 T193D, pcDNA/hSGK2 was used as the template for site-directed mutagenesis using PfuTurbo DNA Polymerase (Agilent Technologies, Santa Clara, CA) and proper mutagenic primers (Supplementary Table 1). To construct pCR3/FLAG/PXR Δ107/141, Δ334/348, pCR3/FLAG/PXR was used as the template for site-directed mutagenesis using PfuTurbo DNA Polymerase and proper mutagenic primers (Supplementary Table 1). After amplification, template was eliminated by digestion with *Dpn*I endonuclease. To construct pcDNA/FLAG/SGK2, pcDNA/FLAG/SGK2 T193A and pcDNA/FLAG/hSGK2 T193D, the full-length cDNAs were amplified with proper PCR primers (Supplementary Table 1), PfuTurbo DNA polymerase and pcDNA/hSGK2, pcDNA/SGK2 T193A and pcDNA/SGK2 T193D, respectively, as the templates. Purified PCR products were cloned into *Eco*RI/*Bam*HI-digested pcDNA/FLAG vector to add FLAG tag. Template DNA was then eliminated by DpnI endonuclease digestion. To construct pGL3/hPepck1 IRS, Pepck1 promoter (–669 to +64) were amplified from HepG2 cells genomic DNA. The amplified DNA fragments were cloned into *Kpn*I/*Xho*I-digested pGL3-Basic. The DNA sequences of these mutants were verified using BigDye Terminator v3.1 Cycle Sequencing Kit (Applied Biosystems, Foster City, CA) and proper pairs of primers.

### Quantitative Real-time PCR (qRT-PCR)

Total RNAs were isolated using TRIzol reagent (Invitrogen), from which cDNAs were synthesized using the MultiScribe Reverse Transcriptase (Applied Biosystems). Real-time PCR was performed using an ABI prism 7700 sequence detection systems (Applied Biosystems). Assays-on-Demand probes (Applied Biosystems) for PCR with the TaqMAN PCR Master Mix (Applied Biosystems) were: human *CYP3A4* gene, Hs00604506_m1; human *PEPCK1* gene, Hs00159918_m1; human *G6Pase* gene, Hs00609178_m1; mouse *PEPCK1* gene, Mm00440636_m1. The TaqMan human and mouse glyceraldehyde-3-phosphate dehydrogenase (Applied Biosystems) was used as internal control.

### siRNA knockdown

siRNA was transfected using Lipofectamine RNAiMAX (Invitrogen); ON-TARGETplus SMART pool human SGK2 siRNA, human NR1I2 siRNA, human PPM1A siRNA and ON-TARGETplus Non-Targeting pool (Dharmacon Research, Lafayette, CO) according to the manufacturer’s instructions. These siRNA-transfected cells were treated with simvastatin (10 μM) for 24 h and 2.5–3 h in ShP51 cells and hepatocytes, respectively. Efficiency of knockdowns was confirmed by qRT-PCR as well as Western blots.

### Luc reporter assays

ShP51 cells were transfected with pGL3/hPepck1 IRS reporter plasmid using Fugene 6 (Promega, Fitchburg, WI) for 24 h. Then cells were exposed to simvastatin (10 μM) for 24 h. Luciferase activities were measured using a Dual-Luciferase reporter assay system (Promega) and normalized against Renilla reniformis luciferase activities.

### Immunoprecipitation

ShP51 cells were transiently co-transfected with pCR3/FLAG/PXR and pcDNA/SGK2, pcDNA/SGK2 T193A and pcDNA/SGK2 T193D or pcDNA/FLAG/SGK2 for 48 hours utilizing Lipofectamine 2000. Similarly, HepG2 cells were transiently transfected with pCR3/FLAG/PXR or constructed PXR deletion mutants and pcDNA/SGK2. These transfected cells were treated with simvastatin (10 μM), pravastatin (10 μM), rifampicin (100 μM) or DMSO for given times, lysed in Buffer A (10 mM HEPES pH 7.6, 10 mM KCl, 1.5 mM MgCl, 0.3% NP-40) containing protease inhibitor and phosphatase inhibitor cocktails and homogenized by Dounce tissue grinder. After centrifugation, cytosols were obtained, diluted in IP lysis buffer (50 mM Tris-HCl pH 7.4, 150 mM NaCl, 1 mM EDTA, 1% Triton X-100) and used for immunoprecipitation. To immunoprecipitate phosphorylated SGK2 protein, cells were boiled in 1% SDS boiling buffer (10 mM Tris-HCl buffer pH 7.4 containing 1% SDS, 1.0 mM sodium ortho-vanadate, protease inhibitors and phosphatase inhibitor cocktails) for 5 min, sonicated and diluted with IP lysis buffer, and used for immunoprecipitation.

### Western blots

Protein extracts were prepared in a LDS sample buffer (78.7 mM Tris-HCl pH 6.8, 2% SDS, 12.5% glycerol). Protein concentrations were determined by Bio-Rad protein assay (Bio-RAD, Hercules, CA), and they were separated on a SDS-PAGE and transferred onto PVDF membranes (GE Healthcare, Pittsburgh, PA). These membranes were blocked with 5% BSA or 5% skim milk in 10 mM Tris-HCl-buffered saline containing 0.1% Tween-20 (TBS-T), incubated overnight at 4 °C with given primary antibodies, washed with TBS-T, incubated with HRP-conjugated secondary antibodies and visualized using ECL plus Western blotting detection reagents (GE Healthcare).

### *In vitro* dephosphorylation assays

SGK2 substrates were prepared using ANTI-FLAG M2-Agarose affinity gels from cytosols of pcDNA/FLAG/SGK2-transfected ShP51 cells with DMSO or simvastatin (10 μM) for 1 h. After washing with IP lysis buffer and dephosphorylation buffer (20 mM Tris, 10 mM MgCl_2_, 1 mg/mL BSA, 0.02% (w/v) Brij-35, pH 7.5), these affinity gels were reacted with recombinant human PP2Cα in dephosphorylation buffer for 30 min at 37 °C. The reaction was terminated by adding a LDS sample buffer, and the resulting lysates were subjected to Western blot analysis with an anti phospho-Thr193 peptide antibody.

### Chromatin immunoprecipitation (ChIP) assays

ChIP assays were performed by using a ChIP-IT Express kit (Active motif, Carlsbad, CA) according to the manufacturer’s instructions. ShP51 cells transfected by a given plasmid or human primary hepatocytes were treated with simvastatin (10 μM) or rifampicin (100 μM) for given times. These cells were cross-linked by formaldehyde. Pellet of these cross-linked cells were lysed, sonicated to shear chromatin DNA and subjected to immunoprecipitated by anti-PXR antibody, anti-SGK2 antibody, anti-RXR antibody, anti-FLAG antibody or a normal mouse IgG at 4 °C for overnight. Immunoprecipitates were washed and eluted. After de-cross-linked and protease-digested, these final DNA samples were PCR-amplified with proper pairs of primers (Supplementary Table 2) and were analyzed by an agarose gel electrophoresis as well as DNA sequencing.

### Statistical Analysis

Data were analyzed with Student’s t test or one-way ANOVA by using GraphPad Prism6 software. A statistical probability of P < 0.05 was considered significant.

## Additional Information

**How to cite this article**: Gotoh, S. and Negishi, M. Statin-activated nuclear receptor PXR promotes SGK2 dephosphorylation by scaffolding PP2C to induce hepatic gluconeogenesis. *Sci. Rep.* **5**, 14076; doi: 10.1038/srep14076 (2015).

## Supplementary Material

Supplementary Information

## References

[b1] Kliewer S. A. *et al.* An orphan nuclear receptor activated by pregnanes defines a novel steroid signaling pathway. Cell 92, 73–82 (1998). 9489701 10.1016/s0092-8674(00)80900-9

[b2] Nakamura K., Moore R., Negishi M. & Sueyoshi T. Nuclear pregnane X receptor cross-talk with FoxA2 to mediate drug-induced regulation of lipid metabolism in fasting mouse liver. J. Biol.Chem. 282, 9768–9776 (2007). 17267396 10.1074/jbc.M610072200 PMC2258557

[b3] Bhalla S., Ozalp C., Fang S., Xiang L. & Kemper J. K. Ligand-activated pregnane X receptor interferes with HNF-4 signaling by targeting a common coactivator PGC-1a. Functional implications in hepatic cholesterol and glucose metabolism. J. Biol. Chem. 279, 45139–45147 (2004). 15322103 10.1074/jbc.M405423200

[b4] Kodama S., Koike C., Negishi M. & Yamamoto Y. Nuclear receptors CAR and PXR cross talk with FOXO1 to regulate genes that encode drug-metabolizing and gluconeogenic enzymes. Mol. Cell. Biol. 24, 7931–7940 (2004). 15340055 10.1128/MCB.24.18.7931-7940.2004 PMC515037

[b5] Kodama S., Moore R., Yamamoto Y. & Negishi M. Human nuclear pregnane X receptor cross-talk with CREB to repress cAMP activation of the glucose-6-phosphatase gene. Biochem J 407, 373–381 (2007). 17635106 10.1042/BJ20070481 PMC2275060

[b6] Bhalla S., Ozalp C., Fang S., Xiang L. & Kemper J. K. Ligand-activated pregnane X receptor interferes with HNF-4 signaling by targeting a common coactivator PGC-1alpha. Functional implications in hepatic cholesterol and glucose metabolism. J. Biol. Chem. 279, 45139–45147 (2004). 15322103 10.1074/jbc.M405423200

[b7] Gotoh S. & Negishi M. Serum- and glucocorticoid-regulated kinase 2 determines drug-activated pregnane X receptor to induce gluconeogenesis in human liver cells. J. Pharmacol. Exp.Ther. 348, 131–140 (2014). 24204015 10.1124/jpet.113.209379 PMC3868883

[b8] Kobayashi T., Deak M., Morrice N. & Cohen P. Characterization of the structure and regulation of two novel isoforms of serum- and glucocorticoid-induced protein kinase. Biochem. J 344, 189–197 (1999). 10548550 PMC1220630

[b9] Sattar N. & Taskinen M. R. Statins are diabetogenic—myth or reality? Atheroscler. Suppl. 13, 1–10 (2012). 22818818 10.1016/j.atherosclerosissup.2012.06.001

[b10] Sukhija R. *et al.* Effect of statins on fasting plasma glucose in diabetic and nondiabetic patients. J. Investig. Med. 57, 495–499 (2009). 10.2310/JIM.0b013e318197ec8b 19188844

[b11] Rysä J. *et al.* Pregnane X receptor agonists impair postprandial glucose tolerance. Clin. Pharmacol. Ther. 93, 556–563 (2013). 23588309 10.1038/clpt.2013.48

[b12] Takasu N. *et al.* Rifampicin-induced early phase hyperglycemia in humans. Am. Rev. Respir. Dis. 125, 23–27 (1982). 7039435 10.1164/arrd.1982.125.1.23

[b13] Carter A. A. *et al.* Risk of incident diabetes among patients treated with statins: population based study. BMJ. 347, f2616, 10.1136/bmjf2610 (2013). PMC3662830 23704171

[b14] Brault M., Ray J., Gomez Y. H., Mantzoros C. S. & Daskalopoulou S. S. Statin treatment and new-onset diabetes: a review of proposed mechanisms. Metabolism 63, 735–745 (2014). 24641882 10.1016/j.metabol.2014.02.014

[b15] Kodama S. & Negishi M. Pregnane X Receptor PXR Activates the GADD45β Gene, Eliciting the p38 MAPK Signal and Cell Migration. J. Biol. Chem. 286, 3570–3578 (2011). 21127053 10.1074/jbc.M110.179812 PMC3030361

[b16] Sherk A. B. *et al.* Development of a small-molecule serum- and glucocorticoid-regulated kinase-1 antagonist and its evaluation as a prostate cancer therapeutic. Cancer Res. 68, 7475–7483 (2008). 18794135 10.1158/0008-5472.CAN-08-1047 PMC2562281

[b17] Howe K., Sanat F., Thumser A. E., Coleman T. & Plant N. The statin class of HMG-CoA reductase inhibitors demonstrate differential activation of the nuclear receptors PXR, CAR and FXR, as well as their downstream target genes. Xenobiotica. 41, 519–529 (2011). 21476904 10.3109/00498254.2011.569773

[b18] Kobayashi K. *et al.* Identification of HMG-CoA reductase inhibitors as activators for human, mouse and rat constitutive androstane receptor. Drug Metab. Dispos. 33, 924–929 (2005). 15802384 10.1124/dmd.104.002741

[b19] Aburai N., Yoshida M., Ohnishi M. & Kimura K. Sanguinarine as a potent and specific inhibitor of protein phosphatase 2C *in vitro* and induces apoptosis via phosphorylation of p38 in HL60 cells. Biosci. Biotechnol. Biochem. 74, 548–552 (2010). 20208361 10.1271/bbb.90735

