# Supplementary material for: Statin-activated nuclear receptor PXR promotes SGK2 dephosphorylation by scaffolding PP2C to induce hepatic gluconeogenesis
Source: Sci Rep. 2015 Sep 22;5:14076. doi: 10.1038/srep14076 (PMC4585725; doi:10.1038/srep14076)

**Supplementary information**

# Statin-activated nuclear receptor PXR promotes SGK2 dephosphorylation by scaffolding PP2C to induce hepatic gluconeogenesis

Saki Gotoh and Masahiko Negishi

**Supplementary Figure 1. Statin induction of the *G6Pase* gene mediated by SGK2 and PXR.** (A) Relative expression of G6Pase mRNA levels measured by qRT-PCR in SGK2 siRNA (left) or PXR siRNA (right)-transfected human primary hepatocytes treated with simvastatin (Simva, 10 μM) for 24 h. Results are shown as fold change relative to DMSO treated control siRNA transfected cells. (n = 3, mean ± s.d.) *P < 0.05, ***P < 0.001. NS, not significant, determined by One-way ANOVA. (B) Relative expression of G6Pase mRNA levels measured by qRT-PCR in SGK2 siRNA (left) or PXR siRNA (right)-transfected ShP51 cells treated with simvastatin (Simva, 10 μM) for 2.5 and 5 h. Results are shown as fold change relative to DMSO treated control siRNA transfected cells. (n = 3, mean ± s.d.) **P < 0.01, ***P < 0.001. NS, not significant, determined by One-way ANOVA.

**Supplementary Figure 2. Effect of phosphatase inhibitors on simvastatin induction of gluconeogenic genes.** Relative expression of PEPCK1 (left) and G6Pase (right) mRNA levels measured by qRT-PCR in ShP51 cells which are pre-treated with (A) sanguinarine chloride (3 μM), (B) okadaic acid (10 nM) or (C) tautomycin (20 nM) and fostriecin (100 nM) for 30 min, followed by co-treatment with simvastatin (Simva, 10 μM) for additional 3

h. Results are shown as fold change relative to DMSO treated cells. (n = 3, mean ± s.d.) **P < 0.01, ***P < 0.001. NS, not significant, determined by One-way ANOVA. (D) Immunoprecipitation of FLAG-SGK2 and Western blot analysis of PP2Cα, PP1α, PP2A and FLAG-SGK2 in pcDNA/FLAG/SGK2 T193A or pcDNA/FLAG/SGK2 T193D-transfected ShP51 cells treated with simvastatin (Simva, 10 μM) for 60 min.

**Supplementary Figure 3. SGK2 binding sites at 107/141 and 334/348 of PXR.** Top, immunoprecipitation of FLAG-PXR WT and FLAG- PXR Δ107/141, Δ334/348 and Western blot analysis of SGK2, PP2Cα and FLAG-PXR in pcDNA/SGK2 and pCR3/FLAG/PXR or pCR3/FLAG/PXR Δ107/141, Δ334/348 transfected HepG2 cells treated with simvastatin (Simva, 10 μM) for 60 min. Bottom, schematic representation of domain structure of human PXR WT and PXR Δ107/141, Δ334/348. DBD: DNA binding domain, LBD: ligand binding domain.

**Supplementary Figure 4. Scheme of identification of PSRE within the upstream region of gluconeogenic genes.** 10 K upstream region of human *G6Pase* gene was searched for PXR binding sequences analyzed by GCG Seqlab. 27 putative binding sites

were aligned with 10 K upstream region of human *PEPCK1*gene, resulting in 5 putative sites found. ChIP assays were subjected to examine PXR levels at regions A (-8,460/-8,033), B (-7,146/-6,824), C (-5,083/-4,633) and D (-1,759/-1,590, PSRE) of the human *G6Pase* promoter in ShP51 cells treated with rifampicin (10 μM) for 1 h.

**Supplementary Figure 5. Statin-treatment increase the human *PEPCK1* IRS promoter in ShP51 cells.** Luciferase activity of human *PEPCK1* promoter in ShP51 cells exposed to simvastatin (Simva, 10 μM) for 24 h. Results are shown as fold change relative to DMSO treated cells (n = 3, mean ± s.d). **P < 0.01, determined by Student's t test.

**Supplementary Figure 6. Statin-treatment did not affect phosphorylated status of SGK2 in mouse livers.** Left, relative expression of PEPCK1 mRNA levels measured by qRT-PCR in livers of simvastatin (50 mg/kg)-administrated mice. Results are shown as fold change relative to control mouse livers (n = 3, mean ± s.e.) NS, not significant, determined by Student's t test. Right, Western blot analysis of p-SGK2, SGK2 and β-actin from whole cell lysates from livers of simvastatin (50 mg/kg)-administrated mice.

**Supplementary Table 1** Primer sequences used for plasmid constructions

| | **Primer Sequences (5'-3')** |
|---|---|
| hSGK2 full length | ACCATGAACTCTAGCCCAGCTGGGACCC;<br>CTAGCAATCCAAGATGTCATCATCCTCTGG |
| Mutation of SGK2 T93A | TGAAGACACCACATCCGCATTCTGTGGTACCCC;<br>GGTACCACAGAATGCGGATGTGGTGTCTTCA |
| Mutation of SGK2 T193D | TGAAGACACCACATCCGACTTCTGTGGTACCCC;<br>GGGGTACCACAGAAGTCGGATGTGGTGTCTTCA |
| FLAG-SGK2 | AACCCAGAATTCAATGAACTCTAGCCCAGCTGGGA;<br>CGTGCTGGATCCCTAGCAATCCAAGATGTCATCA |
| PXR Δ107/141 | CGCAAGTGCCTGGAGAGCGGCATGGGGCTGAC;<br>CATCCGCTGCTCCTCTGTCAGCCCCATGCCGC |
| PXR Δ334/348 | TTCCACTACATGCTGAAGAAGCTGGAGGAGGA;<br>CTGCAGCTGGTCCACCACGCGGTGATGCAGCT |

**Supplementary Table 2** Primer sequences used for ChIP assays

| | **Primer Sequences (5'-3')** |
|---|---|
| PEPCK1 PSRE | TGACACCTGAGAGGTGGCCCT; TGCAGGAGGGGGCCAGACAG |
| PEPCK1 IRS | CCCAAGTTAGGGTGCATCCTTCCCA; ACAGGCAGGTGGGTCAAGGACA |
| G6Pase A | AGGCCGAGGCGGGTAGATCG; TTACAGGCACGCGCCACCAC |
| G6Pase B | CCTCTGCCTCTGTAGTGCGCC; CCTGCCTCAGCCTCCCGAGT |
| G6Pase C | CCCAGCAGGAGTGCAGTGGC; GTGCAGTGGCTCACGCCTGT |
| G6Pase D (PSRE) | AGACCAGCCTGGGCCGCATA; ACACTTGGGTCACGGAGGACTGT |
| G6Pase IRS | TGTCCTGTGTCTCTGGCCTGGT; TCAGGTCAACCCAGCCCTGATCT |

**Supplementary Table 3** Consensus PXR binding site and PSRE sequences

| | **PSRE Sequences (5’-3’)** |
|---|---|
| Consensus PXR binding site | TGTACTCCGTGACCC |
| G6Pase PSRE | AGTCCTCCGTGACCC |
| PEPCK1 PSRE | GGGTCTGCCATGACT (Complementary sequence is similar.) |

# Supplemental Figure 1

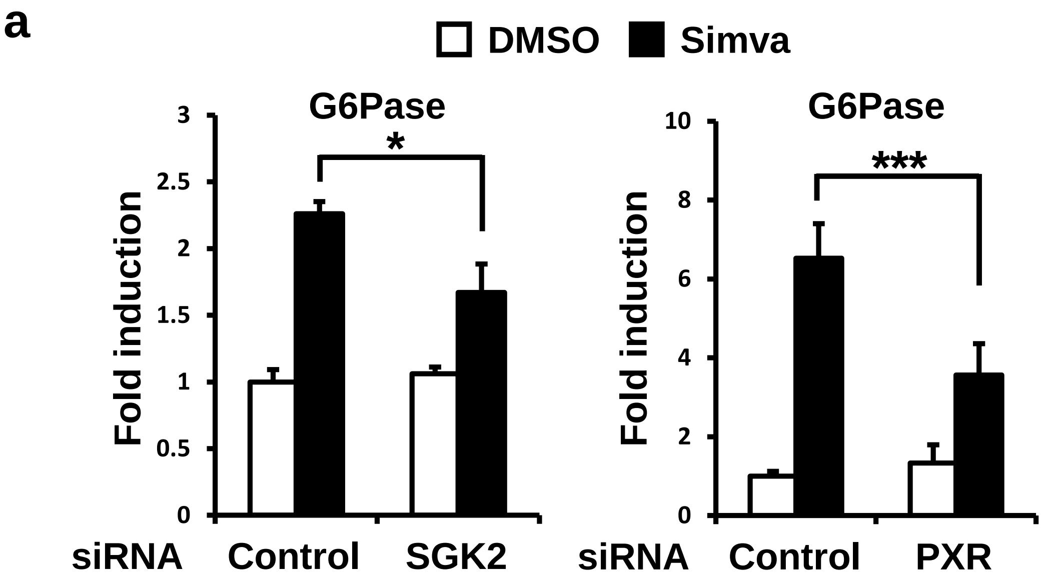


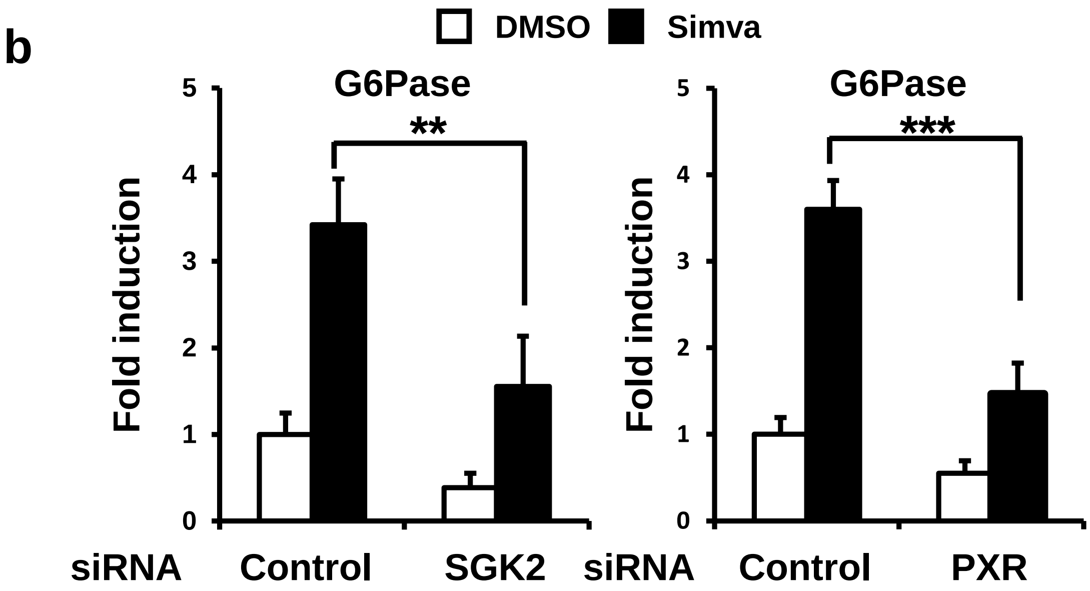

# Supplemental Figure 2

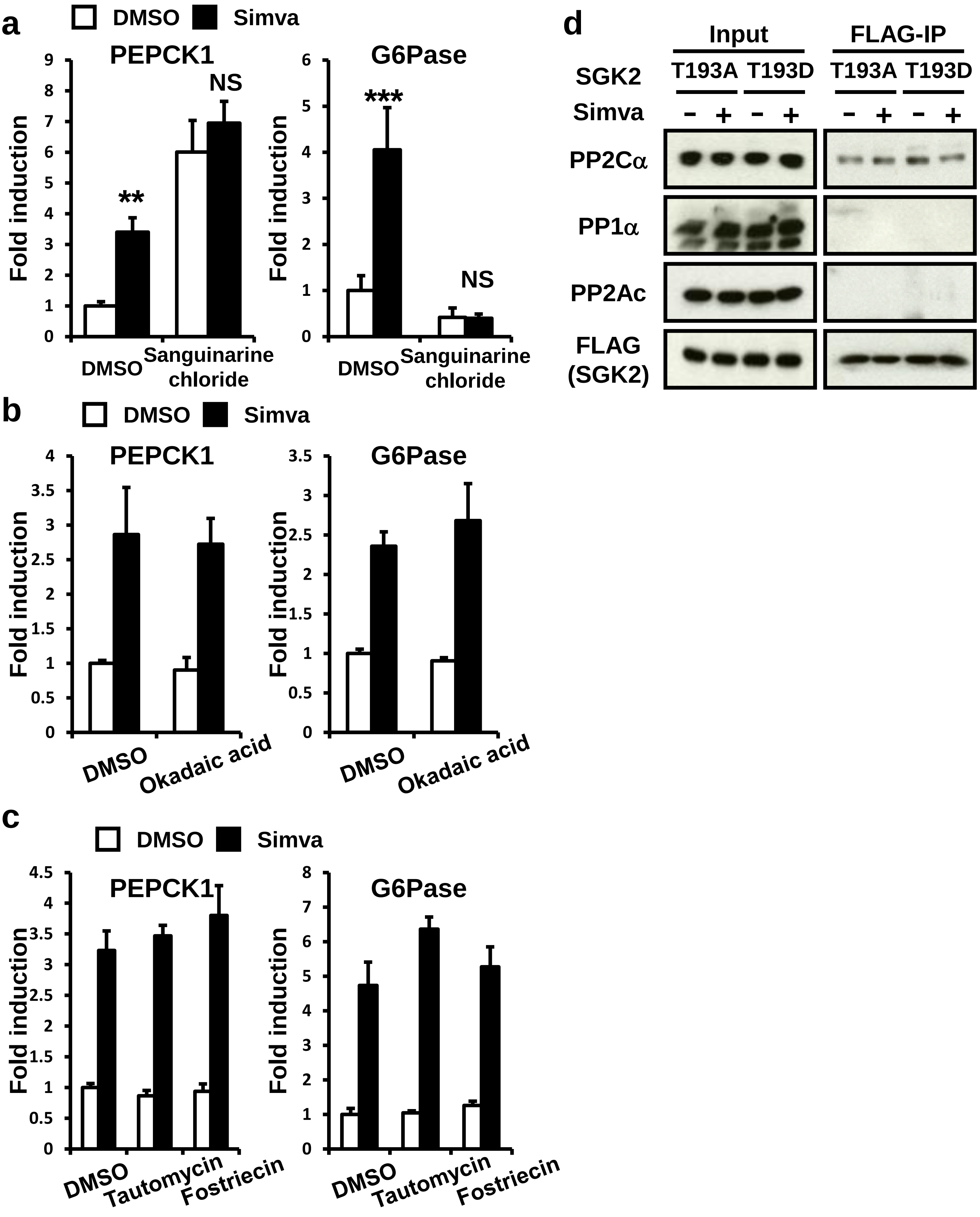

# Supplemental Figure 3

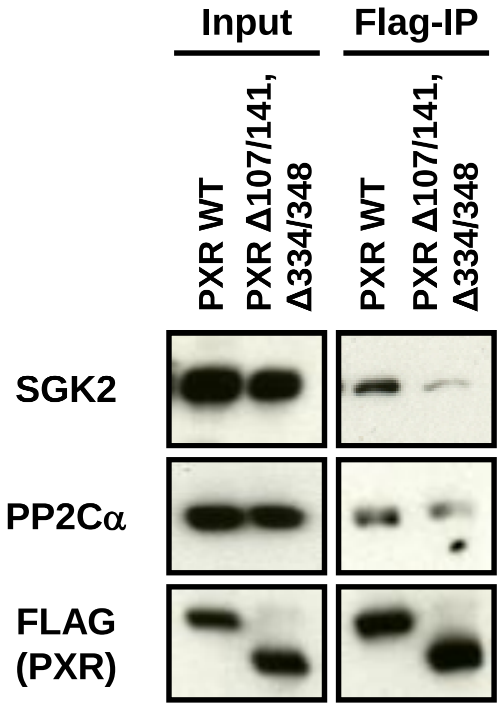


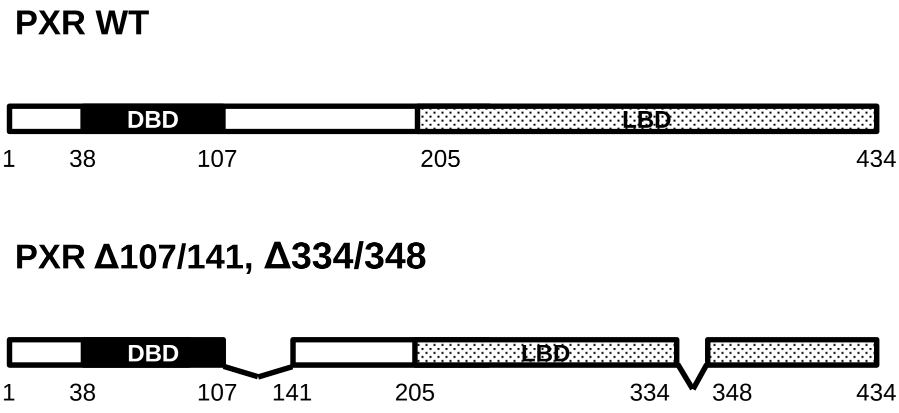

# Supplemental Figure 4

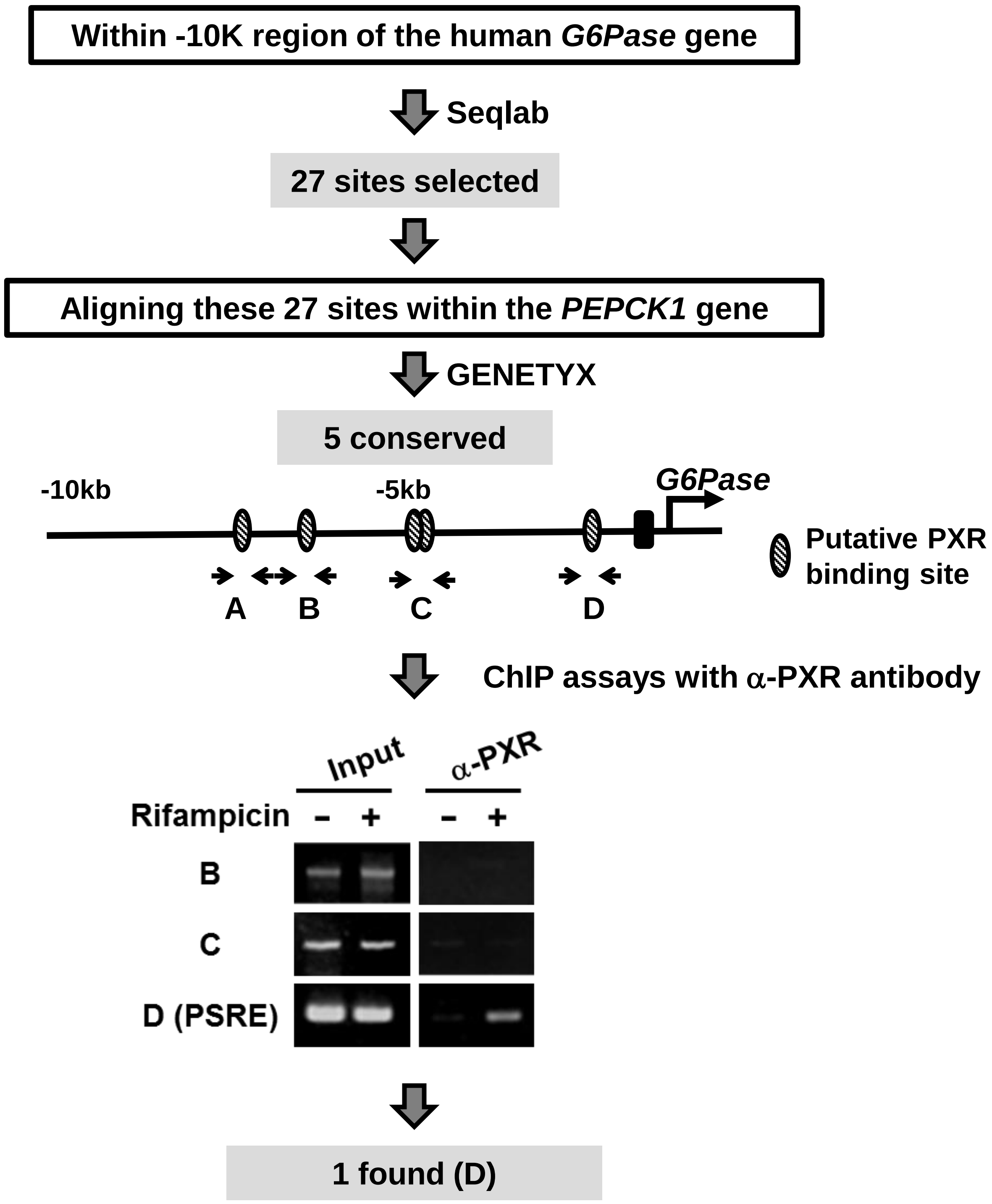

# Supplemental Figure 5

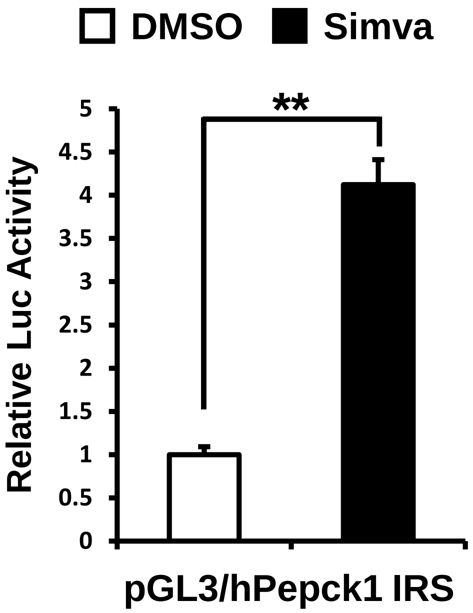

# Supplemental Figure 6

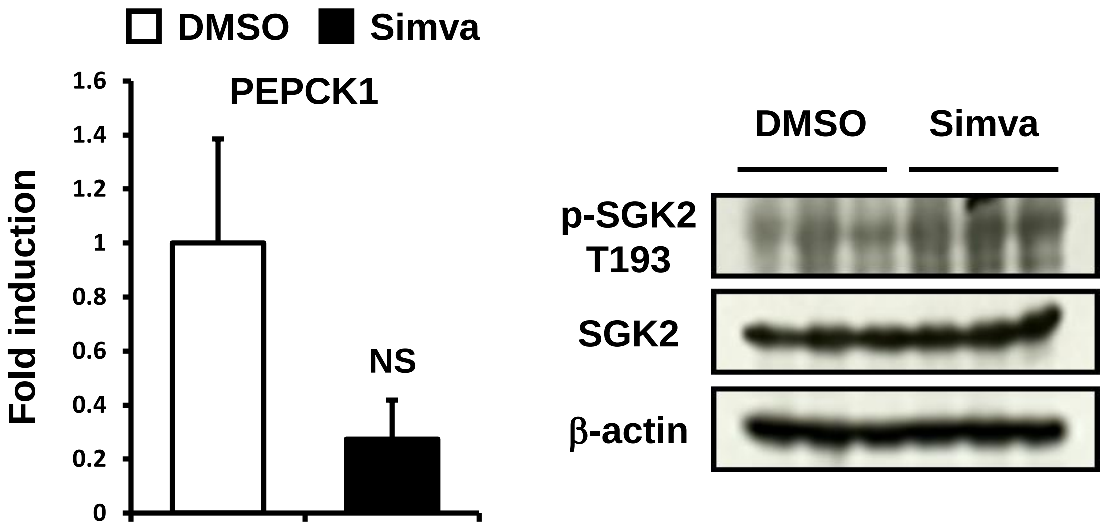

Supplement: Supplementary Information [file srep14076-s1.pdf]
